# Noise pollution effect in flour factory on workers’ hearing in Lamerd City

**Published:** 2015

**Authors:** M Mohammadizadeh, SH Ahmadi, E Sekhavati, K Ahani-Jegar

**Affiliations:** * Department of Environmental Management, Collogue of Natural Resources, Islamic Azad University, Bandar Abbas Branch, Bandar Abbas, Iran,; ** Larestan School of Medical Sciences, Larestan, Iran

**Keywords:** air pollution, workers’ exposure, hearing rate, flour factory

## Abstract

**Introduction:** Noise pollution is one of the most important problems in industry that has an effect on the auditory system and other physiological parameters, as well as persons in noise exposure situations. While noise-induced hearing loss is preventable, once acquired, hearing loss is permanent and irreversible.

**Methodology:** In the current study, noise in various sections of Flour Company in Lamerd estimated via the audio recorder, which revealed that the operators’ expression remained larger than the state criterion; hence, the perception experiment (audio recorder) was performed on the operators and its outcomes were examined via utilizing SPSS 16 of version.

**Findings:** Overall, Pearson relationship r = 0.453 discovered among job reports and the performance decline between all operators by significant stage p≤0.05. Moreover, T-test applied to examine noise impact on operators included in boisterous rooms (mean more than 85 dB) also average=26. 71 and regular deviation=11.72 got (p≤0.05) that was greater than 25db (as the standard hearing threshold).

**Conclusion:** The outcomes of audio measuring and T-test revealed that the noise corruption has an impact on the hearing of bodies operating in noisy rooms.

## Introduction

The requirement for industry in various communities has led to constructing different production factories and industries [**1**]. Environment pollution is a secondary and unwanted product of different industrial activities that has exposed the environment to further danger [**2**]. Noise contamination is one of the extremely significant difficulties of the industries that influence the hearing procedure and other physiological factors of the human’s body, as well as bodies in sound exposure situations [**3**]. This effect and its rate are different according to the personal and environmental features. Important individual characteristics are age, work experience, race, nutrition, and diseases [**4**]. In addition, the exposure to noise can cause social and psychological problems [**5**]. Although noise-induced hearing failure is preventable, already received, the hearing failure is perpetual and immutable [**6**]. About 30 million workers in the USA are exposed to dangerous noise level [**7**,**8**] because of which hundreds of million dollars per year have been estimated for the hearing loss due to noise pollution [**9**]. Statistics of the World Health Organization evaluates 4 million dollars as daily damage [**10**].

One of the industries in which workers are expressed to noise pollution (noise over-limit) because of the presence of noisy machines, is the flour production factory. The presence of mills and huge suction and blower machines made many noise and naturally, it seems that this noise affects the workers’ hearing. 

The position, control, and reduction in the exposure time are regarded as necessary measures. Performing proper control methods can hold the noise of workplace at a standard level [**11**].

Mac Donald’s consulting engineers group investigated the noise pollution in Tehran city in 1977 for the first time, based on which, the noise rate in Tehran city was reported at 55- 85 db [**12**]. 

Investigating the workers’ hearing status in noisy halls of Tehran Azmayesh Factory [**13**], showed that noise has a meaningful impact on the operators’ ears, especially in 4000 Hz frequency; such that only 48.3% of the operators had a normal hearing.

In addition, in a research performed by Qotbi et al. [**14**,**15**] on noise exposure rate and permanent noise-induced hearing of workers of Shadri spinning factory in Yazd city, the results showed that noise and work records have a direct link via their hearing loss.

In a research done on the workers of Taban loom factory in Yazd city, it was revealed that the hearing loss due to NIHL, assuming a constant work record, for a unit increase in the intensity of sound, noise induced hearing loss (NIHL) increased by an average of 0.18.

## Methodology 

At first, resources producing further noise were determined by doing field and library studies about the flour production process in the factory. 

Then all parts of the factory were zoned according to the area and center of each zone, and were determined as a station for measuring noise. Next noise intensity of each station was measured and recorded in network A by using an audiometric device modeled TES-1351 which was calibrated by a calibrator device TES-1352 and totally 234 areas were measured. The average noise of halls and halls with noise over-limit was characterized. 

To do the audiometric test, a screening audiometric device marked Pejvak Ava model ASA 84 was used. 

Personnel were visited three times and before entering the work shift.

**Fig. 1:**
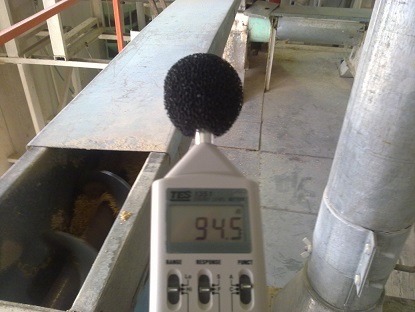
Moment of measuring noise in production hall

Among 29 working persons, an individual was excluded from the study due to having hearing problems related to war, to prevent improper research results. Hearing measurement test was performed for 28 workers while observing the following conditions: 

- Visiting an in and anti acoustic noise room 

- Measuring the hearing rate after rest and before beginning the next work shift 

- Audiometric test in frequencies 500, 1000, 2000, 3000, 4000, 6000, 8000 Hz 

NIHL was determined for any ear individually and for both ears. 

NIHL for both ears was obtained as it follows [**4**]: 

NIHLt=(NIHLb*5)+ (NIHLp)6

NIHL t: general and permanent loss of both ears

NIHLb: permanent loss of strong ear (db)

NIHLp: permanent loss of weak ear (db)

Statistical analysis was done by using SPSS version 16. 

**Findings**

Among 234 measured sections, 41.4% (97 stations) were placed in a hazardous zone and 34.1% (80stattions) in a precaution zone (65- 85 db), 24.3% (57 stations) in a secure zone (lower than 65 db). All the stations of hazardous zone were placed in both production and sifter halls, since there were noisy machines like mills, pull tools, and air compressors. 

Since the legal exposure rate to noise was 85 db for 8 work hours according to the Iran technical protection and professional health committee (obtained from American standard ACGIH) and workers of the factory worked for 8 hours every day. Stations in which noise intense rate was of more than 85 db were determined as areas with noise over-limit (having noise pollution). 

Among 28 people, who have done the hearing test (audiometer), 14 people were showed to noise over-limit level in two production halls and sifter halls and the rest were exposed to noise over-limit level in other sections. 

Measuring the findings of noise for different units in Nasr-e-Lamerd Flour Factory was highlighted in **Table 1** based on the normal limit of noise in Iran. 

**Table 1:** Results of noise measuring in different units of Flour Factory in Lamard based on the legal standard of noise in Iran

Name of unit	Number of station	Danger area		Precaution area		Secure area	
		number	percent	number	percent	number	percent
Production hall	72	66	92	6	8	-	-
Sifter hall	31	31	100	-	-	-	-
Husk hall	72	-	-	72	100	-	-
Administrative unit	14	-	-	1	7	13	93
laboratory	6	-	-	-	-	6	100
Sentry room	9	-	-	-	-	9	100
Support unit	30	-	-	1	3.3	29	96.7
total	234	97	41.4	80	34.1	57	24.3

**Table 2:** Results of noise measuring in different units of Flour Factory in Lamard based on the legal standard of noise in Iran

	Age (years)	Work experience (years)	Noise intensity (db)	NIHL Right ear of all workers	NIHL Left ear of all workers	NIHL two ears of all workers	NIHL two ears of workers having an exposure to more than 85 db
minimum	24	3	56	1.5	0.5	0.9	8.2
maximum	59	21	99.5	48.5	42.5	42.7	42.7
mean	35.8	9.5	82.3	19.3	17.8	17.1	26.5
Standard deviation	7.69	5.4	11.9	14.6	13.6	13.5	11.7

To examine the impact of work record on NIHL, the regression relation was obtained as it follows, with the significance level p value≤0.05: 

NIHL= 3.407+ 5.247* work record

In investigating the link among the work record and NIHL, it was determined that among 5 people worked in very noisy units with work records for lower than 10 years, 4 persons had a normal hearing and 1 person had a partial loss. It seemed that, the noise effect on that person was more intense than on his coworkers because of his age (49 years old). It is deserving considering that the outcome of age on hearing was considered in calculating the NIHL. 

Another point was that, among 9 people working in noisy units (production and sifter halls) with 10 years experience and more, 7 people (78%) had a partial hearing loss, 1 person (11%) had a moderate hearing loss and 1 person (11%) had a normal hearing level. During the study, we realized that a person with a normal range of hearing used earplug regularly; however, the other workers of these 2 units did not use earplugs regularly.

In total, the Pearson correlation r=0.453 was done between the work record and the hearing failure between all operators via a significance stage p≤0.05. 

The studies indicated that, there is a meaningful association among the all workers and NIHL. Pearson relation factor was r= 0.394 in p≤0.05.

The Pearson correlation coefficient was r=0.646 between the age and NIHL in workers of production unit with a significance level p≤0.05, in whom the average noise was of 85 db for 8 working hours. 

The Pearson correlation coefficient was r=0.552 between the age and NIHL in workers of production and sifter units with a significance level p≤0.05 in which the average noise was of 85 db for 8 working hours. 

To investigate the noise effect on the working personnel in noisy rooms (mean of more than 85 db), t-test was used and the mean 26.71 and normal deviation of 11.72 were obtained (p≤0.05). These figures represented more than 25 db (minimum average of people that did not experience any hearing damage) and this showed the noise impact on hearing of people operating in noisy rooms. Also, this average was of 7.08 in other workers who worked in units with an average noise of less than 85 db. 

## Discussion and conclusion

The maximum noise rate was in production and the sifter halls and other halls were less noisy according to the path of these two halls. The audiometric findings revealed that the mean of hearing loss in workers of two noisy units was higher than the other units and the total average, which indicated the link among the increase of environment noise intensity and reduction of hearing rate. Also, there is a clear link among age and work record of people working in the Flour Factory of Lamrard and NIHL; while the relation of its results via the other researches in this area supported this matter. Researches done by Mohammad Heydarian Moghaddam [**16**] indicated that, there is a direct relationship between age, work record, and daily work hours with a decrease in the hearing rate. Parviz Poorkhanzadeh [**17**] also indicated that there is a clear link among the hearing loss and the rise of noise intensity and increase of exposure time, which our research results supported. In a study by Santana and Ferrite, a direct clear link is obtained between age, hearing loss, and occupational exposure to noise [**18**]. A research of Hong and Kim indicated that, there is a meaningful relation between the occupational exposure to noise and the hearing loss [**19**]. The relation between the hearing reduction and age and work experience has been proven in a study done in Ethiopia [**20**], however in the research of Fariba Asghari et al. [**21**], no strong relationship was seen between the hearing loss and work experience and there might be various reasons according to various elements in noise effect. 

This research is the outcome of M.A. thesis in Islamic Azad University of Bandar Abbas. 

**Acknowledgement**

Finally, we deem it necessary to thank the manager and workers of Nasr-e-Lamerd Flour Factory and the experts of the Health Center in Lamerd for their sincere assistance and cooperation in doing this study.
